# Identifying comorbidities and lifestyle factors contributing to the cognitive profile of early Parkinson’s disease

**DOI:** 10.1186/s12883-021-02485-1

**Published:** 2021-12-08

**Authors:** Saul Martínez-Horta, Helena Bejr-Kasem, Andrea Horta-Barba, Berta Pascual-Sedano, Diego Santos-García, Teresa de Deus-Fonticoba, Silvia Jesús, Miquel Aguilar, Lluis Planellas, Juan García-Caldentey, Nuria Caballol, Bárbara Vives-Pastor, Jorge Hernández-Vara, Iria Cabo-Lopez, Lydia López-Manzanares, Isabel González-Aramburu, Maria Asunción Ávila-Rivera, Maria Jose Catalán, Luis Manuel López-Díaz, Victor Puente, Jose Manuel García-Moreno, Carmen Borrué, Berta Solano-Vila, Maria Álvarez-Sauco, Lydia Vela, Sonia Escalante, Esther Cubo, Francisco Carrillo-Padilla, Juan Carlos Martínez-Castrillo, Pilar Sánchez-Alonso, Maria Gema Alonso-Losada, Nuria López-Ariztegui, Itziar Gastón, Marta Blázquez-Estrada, Manual Seijo-Martínez, Javier Rúiz-Martínez, Caridad Valero-Merino, Monica Kurtis, Oriol de Fábregues-Boixar, Jessica González-Ardura, Cristina Prieto-Jurczynska, Pablo Martinez-Martin, Pablo Mir, Jaime Kulisevsky, COPPADIS Study Group

**Affiliations:** 1 Movement Disorders Unit, Neurology Department, Hospital de la Santa Creu i Sant Pau, Mas Casanovas 90, 08041 Barcelona, Spain; 2 Biomedical Research Institute Sant Pau (IIB-Sant Pau), Barcelona, Spain; 3 Centro de Investigación en Red-Enfermedades Neurodegenerativas (CIBERNED), Madrid, Spain; 4 CHUAC, Complejo Hospitalario Universitario de A Coruña, A Coruña, Spain; 5 Complejo Hospitalario Universitario de Ferrol (CHUF), Ferrol, A Coruña, Spain; 6 Unidad de Trastornos del Movimiento, Servicio de Neurología y Neurofisiología Clínica, Instituto de Biomedicina de Sevilla, Hospital Universitario Virgen del Rocío/CSIC/Universidad de Sevilla, Sevilla, Spain; 7 Hospital Universitari Mutua de Terrassa, Terrassa, Barcelona, Spain; 8 Hospital Clínic de Barcelona, Barcelona, Spain; 9 Hospital Quiron Palmaplanas, Clínica Rotger y Centro Neurológico Oms 42, Palma de Mallorca, Spain; 10 Consorci Sanitari Integral, Hospital Moisés Broggi, Sant Joan Despí, Barcelona, Spain; 11 Hospital Universitario Son Espases, Palma de Mallorca, Spain; 12 Neurology Department and Neurodegenerative Diseases Research Group, Vall D’Hebron Universitari Campus, Barcelona, Spain; 13 Complejo Hospitalario Universitario de Pontevedra (CHOP), Pontevedra, Spain; 14 Hospital La Princesa, Madrid, Spain; 15 Hospital Universitario Marqués de Valdecilla, Santander, Spain; 16 Hospital Universitario Clínico San Carlos, Madrid, Spain; 17 Hospital Da Costa de Burela, Lugo, Spain; 18 Hospital del Mar, Barcelona, Spain; 19 Hospital Universitario Virgen Macarena, Sevilla, Spain; 20 Hospital Universitario Infanta Sofía, San Sebastián de los Reyes, Madrid, Spain; 21 Hospital Universitari Josep Trueta y Hospital Santa Caterina, Girona, Spain; 22 Hospital General Universitario de Elche, Alicante, Spain; 23 Fundación Hospital de Alcorcón, Madrid, Spain; 24 Hospital de Tortosa Verge de la Cinta (HTVC), Tortosa, Tarragona, Spain; 25 Complejo Asistencial Universitario de Burgos, Burgos, Spain; 26 Unidad de Trastornos del Movimiento, Servicio de Neurología y Neurofisiología Clínica. Hospital Universitario de Canarias, Tenerife, Spain; 27 Hospital Ramón y Cajal, Madrid, Spain; 28 Hospital Universitario Puerta de Hierro, Majadahonda, Madrid, Spain; 29 Hospital Álvaro Cunqueiro, Complejo Hospitalario Universitario de Vigo (CHUVI), Vigo, Spain; 30 Servicio de Neurología, Complejo Hospitalario Universitario de Toledo, Toledo, Spain; 31 Complejo Hospitalario de Navarra, Pamplona, Spain; 32 Hospital Universitario Central de Asturias, Oviedo, Spain; 33 Hospital Universitario Donostia, San Sebastián, Spain; 34 Servicio de Neurología, Hospital Arnau de Vilanova, Valencia, Spain; 35 Hospital Ruber Internacional, Madrid, Spain; 36 Hospital Lucus Augusti, Lugo, Spain; 37 Hospital Rey Juan Carlos, Madrid, Spain; 38 Centro Nacional de Epidemiología y CIBERNED, Instituto de Salud Carlos III, Madrid, Spain; 39 Departamento de Medicina, Facultad de Medicina, Universidad de Sevilla, Sevilla, Spain

**Keywords:** Parkinson’s disease, PD-MCI, Cognition, Lifestyle, Coppadis

## Abstract

**Background:**

Identifying modifiable risk factors for cognitive impairment in the early stages of Parkinson’s disease (PD) and estimating their impact on cognitive status may help prevent dementia (PDD) and the design of cognitive trials.

**Methods:**

Using a standard approach for the assessment of global cognition in PD and controlling for the effects of age, education and disease duration, we explored the associations between cognitive status, comorbidities, metabolic variables and lifestyle variables in 533 PD participants from the COPPADIS study.

**Results:**

Among the overall sample, 21% of participants were classified as PD-MCI (*n* = 114) and 4% as PDD (*n* = 26). The prevalence of hypertension, diabetes and dyslipidemia was significantly higher in cognitively impaired patients while no between-group differences were found for smoking, alcohol intake or use of supplementary vitamins. Better cognitive scores were significantly associated with regular physical exercise (*p* < 0.05) and cognitive stimulation (< 0.01). Cognitive performance was negatively associated with interleukin 2 (Il2) (*p* < 0.05), Il6 (*p* < 0.05), iron (*p* < 0.05), and homocysteine (*p* < 0.005) levels, and positively associated with vitamin B12 levels (*p* < 0.005).

**Conclusions:**

We extend previous findings regarding the positive and negative influence of various comorbidities and lifestyle factors on cognitive status in early PD patients, and reinforce the need to identify and treat potentially modifiable variables with the intention of exploring the possible improvement of the global cognitive status of patients with PD.

**Supplementary Information:**

The online version contains supplementary material available at 10.1186/s12883-021-02485-1.

## Introduction

Cognitive impairment has long been considered a late complication of Parkinson’s disease (PD) [1, 2]. However, it is currently accepted that cognitive changes of varying severity can be detected from the onset of the disease, and that a significant proportion of PD patients will develop dementia (PDD) during the course of the illness [1–5].

Among the early and heterogeneous cognitive manifestations of PD, alterations that fulfill criteria for mild cognitive impairment (PD-MCI) are associated with an increased risk of PDD [6–8]. At diagnosis, PD-MCI is found in up to 36% of PD patients. Prevalence increases up to 57% after 3.5 years, and progresses to dementia in around 36% of patients within 4 years of diagnosis [3, 9]. The long-term prevalence of PDD is about 80% [2]. Accordingly, both PD-MCI and PDD are frequent features of PD. However, although both entities -especially dementia- have a devastating impact, treatments to delay or prevent the progression of cognitive deterioration in PD are lacking [10]. Recognizing and understanding the mechanisms underlying the development and progression of cognitive impairment in PD is therefore an urgent unmet need. Identifying potentially modifiable variables that may contribute to the progression of cognitive deterioration could improve clinical practice and benefit cognitive trials.

Among the universe of PD patients who will develop dementia, the range of patterns and trajectories in which the condition will progress is wide. The challenge is to predict which early PD patients are likely to develop incident dementia at a more rapid rate. The severity of cognitive changes occurring in PD has been attributed to several neuropathological mechanisms that influence the extent and the progression of brain damage [11]. Genetic factors such as MAPT haplotypes [12–14], GBA mutations or variants of the nurturin gene [15], age, disease duration, protein misfolding and aggregation, synaptic dysfunction and loss, neuroinflammation, and epigenetics are all known to contribute to cognitive changes in PD [16–20]. However, the association between potentially treatable comorbidities, lifestyle factors and cognitive deterioration has been little explored in large samples of early-PD populations, and even less explored using PD-validated and recommended cognitive instruments [17, 21]. Refining such information could enable the design of strategies to prevent key risk factors, improve therapies, slow the progression dementia, and optimize sample selection for cognitive trials.

For this purpose, we used the basal assessments of the COPPADIS study (COhort of Patients with PArkinson’s DIsease in Spain) [22, 23]. The COPPADIS is an observational, 5-year follow-up, nationwide, multicenter study aimed to further clarify the course of PD. It provides data related to a patient’s lifestyle, the presence of other associated comorbidities, and multiple measurements obtained from plasma. The study was designed to use a global cognitive instrument to assess participants’ cognitive status: the PD-specific Parkinson’s Disease-Cognitive Rating Scale (PD-CRS) [24]. Recent guidelines from the Movement Disorders Society (MDS) include the PD-CRS (as ‘recommended’) among the instruments suitable for PD [21]. Based on a large and representative sample of patients with early-stage PD, our main objectives were to explore the possible contribution of potentially modifiable comorbid variables and lifestyle factors to global cognitive functioning.

### Methods

### Participants and variables

Cross-sectional data from the COPPADIS study (database released on May 2019) were used. Details regarding the methods used in the COPPADIS study can be found in the original publications [22, 23]. From the cross-sectional database of 901 participants (207 healthy controls and 694 PD patients) we included only PD participants for whom the following variables were available: age, disease duration, body weight, L-dopa (LD) daily dose, dopamine agonist (DA) daily dose, and LD equivalent daily dose, Hoehn &Yahr (H&Y) stage [25], the Unified Parkinson’s Disease Rating Scale part III (UPDRS-III) [26], the Neuropsychiatric Inventory (NPI) [27], and the Parkinson’s Disease - Cognitive Rating Scale (PD-CRS) [24]. We also collected data regarding the regularity of physical exercise and cognitive stimulation throughout the previous year. We also recorded any history of hypertension, diabetes, dyslipidemia, cardiopathy, arrythmia, smoking, alcohol consumption and supplementary vitamins intake. Several blood testing variables were also collected: levels of protein s100B, TNF alpha, IL-1, IL-2, IL-6, vitamin B12, methylmalonic acid, homocysteine, CRP, uric acid, ferritin, and iron. We excluded patients with a history of chronic anemia, other neurologic diseases, or severe non-compensated diseases. Written informed consent was obtained from all participants and all procedures were performed in accordance with the standards of the ethics committee at each study site, and in accordance with the 1964 Declaration of Helsinki and its later amendments.

### Statistical analysis

Data are expressed as means ± standard deviations (SDs) for continuous variables and as percentages for the categorical variables. Taking into account the normal distribution of the scores according to the Shapiro-Wilk test, we performed group comparisons using independent *t* tests and analyses of variance (ANOVAs) for continuous variables, Mann–Whitney for ordinal data, and the χ^2^ test for categorical variables.

Descriptive analysis of the clinical and sociodemographic characteristics of the sample was performed. According to the PD-CRS, the sample was grouped as cognitively preserved (PD-NC=PD-CRS total score > 80), PD-MCI (PD-CRS total score < 81 and > 64), and PD-dementia (PDD) (PD-CRS total score < 65) [24, 28]. One-way ANOVA and subsequent post-hoc t-test and chi-square comparisons were performed to assess group differences. Bivariate Pearson’s correlation coefficient analysis, logistic regression analysis and linear regression analysis (Mild = 0.20–0.35; Moderate = 0.36–0.60; and High > 0.60) were used controlling the effect of age, education or other potentially confounding variables to explore possible associations between the variables of interest.

## Results

### Clinical and sociodemographic characteristics

The sample consisted of 533 participants (mean age = 62.5 ± 8.6; mean disease duration = 5.52 ± 6 months; median H&Y stage = 2; IQR = 1–4). Table 1 includes all data regarding the participants´ main clinical and sociodemographic characteristics. Using the PD-CRS, among the total sample we found = 114 (21%) were PD-MCI, and 26 (4.8%) were PDD.

**Table 1:** Clinical and sociodemographic characteristics of the sample

***N*** = 533	Mean ± SD	Range
Age	62.5 ± 8.6	35–75
Disease duration (months)	5.52 ± 6	0–114
UPDRS-III^a^ (off)	21.9 ± 11	2–78
H&Y^b^ (off)	2	1–4
Stage 1	14.7%	–
Stage 1.5	9.2%	–
Stage 2	60%	–
Stage 2.5	7.1%	–
Stage 3	7.8%	–
Stage 4	1.3%	–
LDD^c^	527 ± 420	0–2220
DA^d^ equivalent daily dose	182 ± 172	0–1387
PD-CRS^e^ Total	91.8 ± 15.4	44–135

^a^UPDRS: Unified Parkinson’s Disease Rating Scale; ^b^H & Y: Hoehn and Yahr; ^c^LDD: L-dopa daily dose; ^d^DA: Dopaminergic agonist; ^e^PD-CRS: Parkinson’s Disease – Cognitive Rating Scale

As seen in Table 2, no significant differences were found between cognitive groups regarding disease duration, body weight, or medication. Patients in the PD-MCI and the PDD groups were significantly older than those in the PD-NC group [F(1,532) = 31.8; *p* < 0.001]. No differences were found regarding age between PD-MCI and PDD groups [t(140) = − 0.81; *p* = 0.419]. Regarding education level, the proportion of patients with secondary and university studies was significantly higher in the PD-NC group than in the PD-MCI group (χ^2^ < 0.001). The proportion of patients with secondary and university studies was also higher in the PD-NC group than in the PDD group (χ^2^ < 0.001). No differences in education were found between the PD-MCI and PDD groups (χ^2^ = 0.203).

**Table 2:** Clinical, sociodemographic and assessment values for each cognitive group

	PD-NC (***n*** = 393)	PD-MCI (***n*** = 114)	PDD (***n*** = 26)	***p***-value	Post-hoc
Age	60.8 ± 8.8	67.1 ± 6.4	68.1 ± 8.6	< 0.001	^a^ < 0.001; ^b^0.876
Education					
Primary (%)	32.1	68.4	84.6	< 0.001	–
Secondary (%)	35.1	26.3	15.4	< 0.001	–
University (%)	32.6	5.3	0	< 0.001	–
Disease duration (months)	5.59 ± 6.6	5.42 ± 3.5	4.96 ± 4.3	0.858	^a^0.962; ^b^0.964
Hypertension (% yes)	30.3	45.6	34.6	< 0.05	^a^ < 0.05; 0.212
Diabetes mellitus (% yes)	5.6	15.8	15.4	< 0.005	^a^ < 0.05; ^b^0.613
Dyslipidaemia (% yes)	28.8	39.5	38.5	0.070	^a^ < 0.05; ^b^0.554
Cardiopathy (% yes)	7.9	11.4	3.8	0.340	^a^0.162; ^b^0.221
Arrhythmia (% yes)	4.3	7	3.8	0.486	0.176; ^b^0.475
Smoking (% yes))	9.7	9.6	15.4	0.828	^a^0.347; ^b^0.521
Alcohol (% yes)	21.6	19.3	23.1	0.842	^a^0.347; ^b^0.422
Vitamin supplementation (% yes)	5.9	2.6	7.7	0.338	^a^0.126; ^b^0.232
TSREM (% yes)	37.7	39.5	46.2	0.888	^a^ < 0.818; ^b^0.341
UPDRS-III (off)	21.1 ± 11	23.4 ± 11	27.3 ± 11	0.006	^a^0.117; ^b^0.016
H&Y (off)	1.9 ± 0.5	1.9 ± 0.6	2.2 ± 0.6	0.006	^a^0.608; ^b^0.039
LD equivalent daily dose	506 ± 398	567 ± 462	627 ± 530	0.088	^a^0.382; ^b^0.500
DA equivalent daily dose	179 ± 169	182 ± 184	223 ± 177	0.486	^a^0.986; ^b^0.453
Cognitive stimulation (%)	18.1%	12.3%	11.5%	0.268	^a^0.092; ^b^0.610
Exercise (%)	69.7%	64%	50%	0.077	^a^0.150; ^b^0.135
PD-CRS Total	98.8 ± 10	75.1 ± 4	58.8 ± 5	< 0.001	^a^ < 0.001; ^b^ < 0.001
Fronto-subcortical	70.2 ± 10.4	49.1 ± 5.2	36 ± 6.8	< 0.001	^a^ < 0.001; ^b^ < 0.001
*Immediate verbal memory*	8.6 ± 1.8	6.7 ± 2.1	5.7 ± 1.7	< 0.001	^a^ < 0.001; ^b^0.039
*Sustained attention*	9 ± 1.2	7.4 ± 2	5.2 ± 3	< 0.001	^a^ < 0.001; ^b^ < 0.001
*Working memory*	7.7 ± 2	5.2 ± 2	3.6 ± 2	< 0.001	^a^ < 0.001; ^b^ < 0.001
*Clock draw*	9.4 ± 1.4	8.3 ± 1.4	7 ± 2.3	< 0.001	^a^ < 0.001; ^b^ < 0.001
*Delayed free recall*	6.2 ± 2.5	4.1 ± 2	2.3 ± 2	< 0.001	^a^ < 0.001; ^b^ < 0.001
*Alternating verbal fluency*	12.8 ± 3.7	7.2 ± 2.6	5.5 ± 3.3	< 0.001	^a^ < 0.001; ^b^0.078
*Action verbal fluency*	16.4 ± 5.3	10 ± 3.1	7.6 ± 3.3	< 0.001	^a^ < 0.001; ^b^0.059
Posterior-cortical	28.5 ± 2.6	26 ± 3.6	22.8 ± 4	< 0.001	^a^ < 0.001; ^b^ < 0.001
*Confrontation naming*	18.7 ± 2.4	16.9 ± 3.6	14.8 ± 3.9	< 0.001	^a^ < 0.001; ^b^0.002
*Clock copy*	9.8 ± 1	9 ± 1	8 ± 2	< 0.001	^a^ < 0.001; ^b^ < 0.001
Protein S100B	0.125 ± 0.6	0.094 ± 0.10	0.086 ± 0.01	0.932	^a^0.941; ^b^0.999
TNF alpha	7.2 ± 3	7.5 ± 4.5	6.4 ± 3.5	0.701	^a^0.903; ^b^0.684
IL-1	3.8 ± 15.1	4.4 ± 6.7	6.5 ± 7.3	0.849	^a^0.969; ^b^0.917
IL-2	1.9 ± 7.5	6.4 ± 33.8	30.5 ± 79.4	< 0.005	^a^0.529; ^b<^ 0.005
IL-6	2.3 ± 3.5	4.2 ± 9.6	9 ± 17.7	< 0.01	^a^0.242; ^b<^ 0.05
Vitamin B12	396.4 ± 152.2	395.4 ± 209.9	358 ± 137.48	0.817	^a^0.999; ^b^0.831
Methylmalonic acid	0.16 ± 0.1	0.17 ± 0.1	0.26 ± 0.26	0.070	^a^0.767; ^b^0.168
Homocysteine	14 ± 5.3	15.3 ± 7.7	16.2 ± 4	0.355	^a^0.488; ^b^0.914
CRP	0.2 ± 0.3	0.22 ± 0.29	0.19 ± 0.34	0.922	^a^0.917; ^b^0.974
Uric acid	5.5 ± 2.2	5.5 ± 1.6	5.8 ± 0.9	0.916	^a^0.999; ^b^0.927
Ferritin	126.6 ± 110	117 ± 163	60 ± 58	0.367	^a^0.906; ^b^0.500
Iron	91.7 ± 36.7	80 ± 31.4	80 ± 30.5	0.158	^a^0.174; ^b^0.999

^a^ *p* ≤ 0.05 significance based on paired t-test. (PD-NC vs PD-MCI)

^b^ *p* ≤ 0.05 significance based on paired t-test. (PD-MCI vs PDD)

Bivariate correlation analysis showed a significant moderate association between older age and worse cognitive status (*r* = − 0.410; *p* < 0.001). However, disease duration was not significantly associated with global cognitive performance.

### Cognitive and neuropsychiatric profile

Significant differences were found between groups in the total PD-CRS score [F(1,532) = 430.2; *p* < 0.001] and in related sub-scores. We found a clear pattern of worse performance in the PD-CRS total score in the PD-MCI group with respect to the PD-NC group [t(507) = 34.2; *p* < 0.001], and in the PDD group with respect to the PD-MCI [t(140) = 15.6; *p* < 0.001] and PD-NC [t(419) = 32.1; *p* < 0.001] groups. As depicted in Table 2, we also found significant differences between patients in the PD-NC and PD-MCI groups, and between patients in the PD-MCI and PDD groups in the scores from all the subtests comprising the PD-CRS.

The prevalence of neuropsychiatric symptoms (Table 3) presenting with sufficient severity to be considered clinically relevant (NPI > 1) differed significantly between groups for dysphoria (*p* < 0.05) and for apathy (*p* < 0.005). These differences were driven by an increased prevalence of dysphoria in the PD-MCI group (41.8%) with respect to the PD-NC (28.8%) and PDD groups (33.3%), and by a progressively higher prevalence of apathy in the three groups (PD-NC = 21.4% vs PD-MCI = 35.4% vs PDD = 42.9%). Post-hoc comparisons determined higher scores for dysphoria in the PD-MCI group than in the PD-NC group [t(507) = − 2.17; *p* < 0.05], and higher scores for apathy in the PD-MCI group than in the o PD-NC group [t(507) = − 2.98; *p* < 0.005]. Higher apathy scores were also found in the PDD group with respect to the PD-NC group [t(419) = − 2; *p* < 0.05]. Clinically relevant apathy increased the odds ratio for being classified as PD-MCI by 2 [OR = 2.01; 95% CI = 1.2–3.2] and for being classified as PDD by 2.2 [OR = 2.2; 95% CI = 0.9–5.5].

**Table 3:** Neuropsychiatric symptoms

	PD-NC	PD-MCI	PDD	***p***-value	Post-hoc
**NPI Total (frequency x severity)**	5.4 ± 7.3	7.7 ± 8.7	9 ± 9.9	0.007	^a^ < 0.001; ^b^0.721
% NPI total > 1	60.4	70.2	72.7	0.126	^a^0.05; 0.518
Delirium	0.1 ± 0.9	0.2 ± 0.9	0 ± 0	0.586	^a^0.726; ^b^0.619
% NPI > 1	2.3	4.3	0	0.432	^a^0.246; ^b^0.441
Hallucinations	0.1 ± 0.7	0.3 ± 1.5	0.3 ± 0.8	0.162	^a^0.150; ^b^0.951
% NPI > 1	3.3	7.1	9.5	0.138	^a^0.088; ^b^0.496
Agitation	0.4 ± 1.2	0.5 ± 1.3	0.9 ± 1.7	0.183	^a^0.585; ^b^0.525
% NPI > 1	9.1	13.5	19	0.198	^a^0.141; ^b^0.362
Depression/dysphoria	1.3 ± 2.1	2 ± 2.9	2.2 ± 3.4	0.020	^a^0.034; ^b^0.258
% NPI > 1	21.8	41.8	33.8	0.055	^a^ < 0.05; ^b^0.320
Anxiety	1.4 ± 2.3	1.6 ± 2.3	1.5 ± 2.1	0.708	^a^0.684; ^b^0.988
% NPI > 1	33	39.2	34.8	0.537	^a^0.160; ^b^0.445
Euphoria	0.2 ± 0.9	0.2 ± 0.8	0 ± 0	0.666	^a^0.934; ^b^0.548
% NPI > 1	4.7	6.3	0	0.460	^a^0.340; ^b^0.293
Apathy	0.9 ± 2	1.9 ± 3	2.3 ± 2.7	< 0.001	^a^ < 0.001; ^b^0.839
% NPI > 1	21.4	35.4	42.9	< 0.005	^a^ < 0.005; ^b^0.341
Disinhibition	0.06 ± 0.4	0.1 ± 0.6	0 ± 0	0.183	^a^0.230; ^b^0.341
% NPI > 1	1.7	3.2	0	0.520	^a^0.294; ^b^0.543
Irritability	0.8 ± 1.6	1.1 ± 2	1.3 ± 2.3	0.132	^a^0.207; ^b^0.930
% NPI > 1	20	27.4	27.3	0.265	^a^0.086; ^b^0.610
Aberrant motor behaviour	0.2 ± 1	0.2 ± 0.7	0.7 ± 2.1	0.102	^a^0.985; ^b^0.134
% NPI > 1	4	6.4	9.5	0.373	^a^0.238; ^b^0.448

^a^ *p* ≤ 0.05 significance based on paired t-test. (PD-NC vs PD-MCI)

^b^ *p* ≤ 0.05 significance based on paired t-test. (PD-MCI vs PDD)

### Comorbidities and lifestyle factors

No differences were found between cognitive groups regarding the proportion of patients performing regular physical exercise, or between those performing regular cognitive stimulation during the previous year. However, in the total sample, the PD-CRS total score was significantly associated with the regularity of physical exercise (β = 0.105; *p* < 0.05) and cognitive stimulation (β = 0.120; *p* < 0.01).

The prevalence of hypertension was significantly higher in the PD-MCI group (45.6%) than in the PD-NC (30.3%; *p* < 0.005) and PDD groups (34.6%; *p* < 0.05) and was associated with an increased risk of 1.9 for PD-MCI [OR = 1.9; 95% CI = 1.2–2.9]. The prevalence of diabetes was significantly increased in the PD-MCI (15.8%) and PDD (15.4%) groups with respect to the PD-NC group (5.6%), and it was associated with a three times greater risk for PD-MCI [OR = 3.1; CI = 1.6–6.1] and a two times greater risk for PDD [OR = 2.1; CI = 0.6–6.4]. Dyslipidemia was also significantly increased in both the PD-MCI (39.5%) and the PDD (38.5%; *p* < 0.05) groups with respect to PD-NC group (28.8%). No between-group differences were found for the other comorbidities, including history of smoking, alcohol intake and use of supplementary vitamins. Regarding values collected in blood samples, these were available for the 33.6% of the sample (74.4% PD-NC; 21.2% PD-MCI; 4.3% PDD). Between-group differences were significant for IL-2 [F(1,532) = 6.1; *p* < 0.05] and for IL-6 [F(1,532) = 4.9; *p* < 0.01] levels, and a tendency was found for methylmalonic acid levels [F(1,532) = 2.7; *p* = 0.07]. Post-hoc comparisons determined that these differences were driven by higher IL-2 levels in PDD with respect to both PD-MCI [t(507) = − 4.4; *p* < 0.05] and PD-NC [t(507) = − 28; *p* < 0.005], and by higher IL-6 values in PDD with respect to PD-NC [t(507) = − 6.6; *p* < 0.05].

### Correlates and predictors analysis

Linear regression analysis showed significant associations between higher PD-CRS total score and younger age (β = − 0.416; *p* < 0.001), education level (β = − 0.451; *p* < 0.001) and lower UPDRS-III (β = − 0.102; *p* < 0.05). Significant associations independent of age, education and motor status were also found between the PD-CRS total score and the NPI apathy score (β = − 0.173; *p* < 0.005).

In the bivariate Pearson’s correlation analysis performed in the total sample, the PD-CRS total score was negatively associated in an almost negligible range with the levels of Il2 (*r* = − 0.146; *p* < 0.05), Il6 (*r* = − 0.178; *p* < 0.05), homocysteine (*r* = − 0.172; *p* < 0.05) and iron (*r* = − 0.171; *p* < 0.05). However, these values were also strongly associated with age and LD daily dose. Accordingly, to prevent the potential influence of age and LD, these variables were included as covariates in a linear regression model. This analysis showed a significant association between PD-CRS total score and vitamin B12 values (β = 0.169; *p* < 0.05), homocysteine levels (β = − 0.194; *p* < 0.05) and iron levels (β = 0.177; *p* < 0.05), and a specific mild association between PD-CRS posterior-cortical performance and IL2 values (β = − 0.269; *p* < 0.005).

Logistic regression analysis (step forward conditional) was used to explore the classification ability of the various measures. Along with age, the model included education, UPDRS-III, measures regarding comorbidities, non-motor symptoms, neuropsychiatric symptoms, cognitive performance, lifestyle factors (smoking, alcohol intake, regularity of exercise), and blood-sample measurements. Focusing on the PD-NC and PD-MCI groups, we observed that beyond the PD-CRS, age (β = 0.091; *p* < 0.001), education level (β = − 1.06; *p* < 0.001) and dysphoria (β = 0.058; *p* < 0.01) were the strongest contributors to PD-MCI classification. Regarding the PDD group, the strongest associations were found with age (β = 0.102; *p* < 0.05), education level (β = − 2; *p* < 0.001), UPDRS-III (β = 0.056; *p* < 0.05), and apathy severity (β = 0.197; *p* < 0.05). Regarding blood-sample values, no significant associations were found with PD-MCI. However, Il6 values were associated with PDD (β = 0.076; *p* < 0.05).

## Discussion

Our main findings confirm that metabolic variables such as interleukins, homocysteine, iron, and vitamin B12 have an impact on cognitive status in early PD patients, while physical exercise and cognitive stimulation have a protective effect.

According to the recommended Level I instrument (the PD-CRS), the prevalence of PD-MCI in the COPPADIS sample at baseline was 21%, whereas the prevalence of PDD was 4%. In the main, these prevalences of cognitive impairment are equivalent to those reported in other cohorts of early-stage PD patients [29–31]. Our data thus highlight the number of subjects in a representative population of early PD patients who meet criteria for PD-MCI and even PDD.

As expected, older age was significantly associated with worse cognitive status, but disease duration was not, supporting the idea that disease duration is not necessarily associated with the extent of cognitive deterioration, at least during the early stages of PD, and that other factors intervene in this population.

PD-MCI and PDD were associated with older age and with education level, but no differences were found between PD-NC and PD-MCI groups regarding motor status and disease stage. Conversely, although the range was similar in terms of disease duration, the PDD group scored significantly worse in the UPDRS and had a greater H&Y staging than the PD-NC and PD-MCI groups. This again suggests that other mechanisms, not necessarily related to disease duration only, participate in the exacerbation of PD both at motor and cognitive levels [32–34].

Among the potentially treatable comorbidities, hypertension, diabetes mellitus, and dyslipidemia appeared to be more frequent among patients with PD-MCI and PDD. These variables play a critical role in the development of vascular lesions and alterations of white matter in the general population. This type of injury is usually accompanied by cognitive signs that may trigger cognitive impairment and dementia. In future studies, therefore, it is necessary to emphasize the contribution of brain anomalies secondary to these variables in the development of cognitive impairment in PD. Focusing on lifestyle factors, we found no significant differences between groups in terms of regularity of physical exercise, cognitive stimulation, smoking, or alcohol intake. However, in the overall sample, there was a positive association between the regularity of physical exercise, cognitive stimulation, and global cognitive status. Unfortunately, the data analyzed do not allow us to specify which type of physical and cognitive exercise, or the frequency of such activities, could be potentially beneficial. In this sense, our results justify more detailed future study concerning the potential beneficial effect of physical and cognitive exercise programs.

Evidence suggesting that exercise may positively influence the general management and the cognitive outcomes in PD is increasing. Intervention programs of moderate intensity aerobic exercise 2–3 times per week can lead to long-term improvement in attention/working memory and executive function [35] in non-demented patients with mild-to-moderate PD. The association between higher cognitive scores and regularity of physical exercise found in the present study concurs with this information and supports the implementation of strategies to motivate adherence to regular exercise programs. Although the body of evidence is not large, a small beneficial effect on cognition can still be demonstrated with cognitive training in non-demented PD patients [36–38]. Notably, as is the case of physical exercise, most gains were noted in selected tests of attention, working memory and executive skills, while not improvement was noted in global cognition.

From the values collected in blood samples, interleukins IL-2 and IL-6 levels were significantly increased in the PDD group compared to those in the PD-MCI and PD-NC groups. The association of these values with the LD daily dose regime suggested that these results could be influenced by drug treatment. However, when controlling for this effect we still found a significant association between PD-CRS total score and levels of vitamin B12, homocysteine and iron, and a specific association between Il2 levels and PD-CRS posterior-cortical performance. Both increased homocysteine and decreased vitamin B12 levels have been associated with mild cognitive impairment and risk of progression to Alzheimer’s type dementia, vascular dementia, and cognitive impairment in PD [39–42]. Homocysteine, the metabolic product of methionine, has toxic effects on neurons and vascular walls, and its levels are regulated by several pathways, which in turn are conditioned by factors such as age, genetics, diet, gender and pharmacological treatments. In PD, treatment with LD has been found to contribute to hyperhomocysteinemia [43]. Therefore, it is especially relevant to take into account that in addition to the cardiovascular risk factors that patients with PD may have, treatment has an effect on metabolism that can add complexity to the relationship between PD, cardiovascular pathology and cognitive impairment. In this sense, it is also important to take into account that our results show a relationship between homocysteine levels, vitamin B12 and cognitive status that is independent of age and LD treatment. Therefore, it is necessary to emphasize that abnormalities in homocysteine values in PD may not respond exclusively to the effect dictated by dopaminergic treatment. In any case, taking into account this relationship and that found in previous studies, the need to consider the effect of this variable when we explore cognition in PD and the mechanisms that associate more aggressive cognitive phenotypes is indisputable. Recently, a relationship between levels of iron accumulation and cognitive changes in PD has also been demonstrated [44, 45]. Iron is ubiquitous in numerous normal biological processes and in neurodegeneration [46], and undoubtably, brain iron accumulation exerts significant deleterious effects on cognition. Although the relationship between plasma iron levels and cognition is much less well understood, the reported findings reinforce the need to explore the mechanisms involved in the increase in plasma iron levels and the possible contribution of these levels to cognitive impairment in PD. Both Il2 and Il6 pro-inflammatory cytokines that stimulate the inflammatory and auto-immune processes have been suggested to contribute to apoptosis and neurodegeneration in several diseases, including multiple sclerosis [47], Alzheimer’s disease [48], and PD [49], and to play a possible role in exacerbating cognitive decline in relatively early stages.

The major strength of this study is the use of a large and representative observational sample recruited from different practice settings to establish the influence of modifiable risk factors on the cognitive profile of early-PD patients. A second strength is the use of a PD-specific and recommended cognitive assessment instrument. Some limitations should be acknowledged. The COPPADIS cohort is not a de novo cohort, and patients less than 6 years from diagnosis were enrolled at all levels of cognitive impairment and disease duration. The study does not include data on other potential modifiers of cognitive status such as Mediterranean diet, caloric restriction, or social engagement that were not part of the COPPADIS sample, and it does not explore the effect of the considered variables on the structural integrity of grey matter and white matter tracts.

Undoubtedly, any attempt to mechanistically explain the translation between the values detected in our measurements and their partial effect on the progression of cognitive impairment in PD - which appears to be mainly linked to the multiple processes involved in the pathogenesis of the disease -, could be an oversimplification. Nevertheless, while we await the development of more specific treatments, our data support the need for a global prevention strategy to manage cognitive impairment in early-PD, targeting the identified risk factors. Early identification of cognitive impairment in PD must be carried out using adequate screening instruments. Prevention and treatment of metabolic aspects and other corrective measures, and stronger strategies to motivate practice and adherence to regular exercise and cognitive stimulation programs should be encouraged.

On the basis of our results, future research regarding cognitive impairment in PD should take into account the role of the comorbidities described here. Specifically, it would be especially relevant to explore the cognitive progression profile of the participants in the COPPADIS study based on the variables identified in the present study. Likewise, it would be interesting to explore the brain correlates that could accompany the way in which these variables are expressed. In addition, the effect mediated by comorbidities should be taken into account when designing clinical trials.

## Conclusions

Metabolic variables such as interleukins, homocysteine, iron, and vitamin B12 have an impact on cognitive status in early PD patients, and physical exercise and cognitive stimulation have a protective effect. Beyond the main neuropathological mechanisms.

that influence the progression of cognitive impairment in PD, our data also support the need to consider other frequent comorbidities and variables related to lifestyle when addressing cognitive impairment in PD.

## Supplementary Information

**Additional file 1.**

## Data Availability

The protocol and the statistical analysis plan are available on request from the corresponding author. De-identified participant data are not available for legal and ethical reasons.
